# Does articular and entheseal structural damage drive pain and patient-driven reported outcomes in patients with difficult-to-treat psoriatic arthritis? Results from a cross-sectional study

**DOI:** 10.1016/j.ero.2026.100217

**Published:** 2026-07-16

**Authors:** Fabio Massimo Perrotta, Alen Zabotti, Mauro Fatica, Nicola Cabas, Andrea Guiotto, Nicoletta Franzolini, Ivan Giovannini, Maria De Martino, Cristina Di Nicola, Miriam Isola, Andrea Di Matteo, Gabriele De Marco, Dennis McGonagle, Luca Quartuccio, Ennio Lubrano

**Affiliations:** 1 Academic Rheumatology Unit, Dipartimento di Medicina e Scienze della Salute “Vincenzo Tiberio”, Università degli Studi del Molise, Via Giovanni Paolo II, C/da Tappino, Campobasso, Italy; 2 Department of Medical and Biological Sciences, Rheumatology Clinic, University Hospital Santa Maria della Misericordia, Udine, Italy; 3 Section of Musculoskeletal Disease, NIHR Leeds Musculoskeletal Biomedical Research Centre, Leeds Institute of Rheumatic and Musculoskeletal Medicine, University of Leeds, Chapel Allerton Hospital, Leeds, UK; 4 Presidio Ospedaliero “S. Antonio”, San Daniele (Udine), Italy; 5 Department of Medicine, University of Udine, Udine, Italy; 6 Epidemiology Unit, Italian Society for Rheumatology, Milan, Italy

## Abstract

**Objectives:**

This study aims to assess difficult-to-treat psoriatic arthritis (D2T-PsA) and determine whether articular and entheseal structural damage, assessed by ultrasound (US), may drive pain and patient-driven outcomes in D2T-PsA.

**Methods:**

A multicentre cross-sectional study was conducted on patients with PsA treated with biologic/targeted synthetic disease-modifying antirheumatic drugs (b/tsDMARDs). Eligible participants were required to meet the following inclusion criteria: age ≥18 years; diagnosis of PsA fulfilling the classification criteria for Psoriatic Arthritis (CASPAR); classification as D2T-PsA, defined by failure of ≥2 classes of b/tsDMARDs (with different mechanisms of action) following the failure or contraindication of ≥1 conventional synthetic DMARD, and persistently active disease (Disease Activity Index for PsA >14). Sonographic evaluation was performed by using the US damage score (ultrasound in psoriatic arthritis [UPsA]) from the Ultrasound in PSoriatic arthritis TREAtMent (UPSTREAM) study. We used Spearman rho correlation coefficient to evaluate correlations between UPsA damage score and clinical features.

**Results:**

53 patients with PsA were included in the analysis. The median (IQR) UPsA damage score of the 53 D2T patients was 3.79 (1.28-5.45). The UPsA damage score negatively correlated with tender joint count (rho –0.27, *P* = .04) but positively correlated with swollen joint count (rho +0.27, *P* = .05). The number of joint erosions and joint osteophytes/osteoproliferation correlated positively with physician global assessment of disease activity (PhGA) (rho +0.41, *P* = .002) (rho +0.27, *P* = .04) and correlated negatively with Δ patient global assessment of disease activity (PtGA)-PhGA (rho –0.30, *P* = .02) (rho –0.31, *P* = .02). No significant correlation was detected between pain and PtGA with UPsA damage score.

**Conclusions:**

Preliminary results of our study in patients with D2T PsA showed that articular damage could be associated with PhGA and swollen joints, but not with pain, suggesting that patient-driven outcomes may potentially be related to inflammation or noninflammatory mechanisms rather than damage itself.

## INTRODUCTION

Psoriatic arthritis (PsA) is a chronic inflammatory disease that affects skin and joints with possible association with extra-articular manifestations and comorbidities [1]. Despite the substantial improvement of the treatment strategies in PsA over the past 2 decades, marked by the introduction of numerous biologic disease-modifying antirheumatic drugs (bDMARDs) and targeted synthetic DMARDs (tsDMARDs), achieving remission or low disease activity remains a significant challenge for many patients [2].

In fact, the multidomain nature of PsA, characterised by the involvement of multiple tissues and the high prevalence of comorbidities, further exacerbates the complexity of its management and frequently interferes with therapeutic responses [3,4].

Recently, the group for research and assessment in psoriasis and psoriatic arthritis (GRAPPA) and the European alliance of association for rheumatology (EULAR) task forces have been convened to define this challenging clinical phenotype in PsA which is “difficult-to-treat” [[5], [6], [7]].

In our recent work [8,9], we proposed the potential benefits of distinguishing between 2 distinct clinical phenotypes: persistent inflammatory PsA, characterised by ongoing inflammation confirmed through imaging, and noninflammatory PsA (NIPsA) [8], where there is a discrepancy between patient-reported symptoms and clinical examination findings, which do not reveal clear articular inflammation. Patients with NIPsA may be challenging to manage because the patient-reported symptoms can be driven by several factors [10], including comorbidity and the presence of joint/entheseal structural damage which may increase pain perception and reduce function and quality of life [11]. In this context, the aim of this study was to assess D2T PsA and determine whether articular and entheseal structural damage, assessed by ultrasound (US), may drive pain and patient-driven outcomes.

## METHODS

### Study design and participants

This multicentre, cross-sectional observational study was conducted from May to December 2024 at 2 Italian centres: the Rheumatology Clinic of Santa Maria della Misericordia Hospital (Udine) and the Rheumatology Unit of University of Molise (Campobasso).

Eligible participants were required to meet the following inclusion criteria: (i) age ≥18 years; (ii) diagnosis of PsA fulfilling the classification criteria for Psoriatic Arthritis (CASPAR) [12]; (iii) classification as D2T-PsA, defined by failure of ≥2 classes of b/tsDMARDs (with different mechanisms of action) following the failure or contraindication of ≥1 conventional synthetic DMARD, and persistently active disease (disease activity index for PsA [DAPSA] >14) which must be present at the enrolment visit. [13]; (iv) presence of at least 1 clinically significant site, defined as a tender and/or swollen joint or a tender enthesis for peripheral disease, or axial inflammatory symptoms combined with spine or sacroiliac tenderness for axial disease, detected during physical examination; and (v) ongoing continuous therapy for at least 3 months before enrolment.

Consecutive patients with PsA meeting these criteria underwent comprehensive rheumatologic evaluations and independent, blinded sonographic assessments, both performed on the same day.

### Data collection and rheumatological assessment

For each patient, complete demographic data were recorded (ie, age, sex, smoking status), anthropometric measurements (height and weight), as well as medical history regarding comorbidities. Each patient underwent an extended rheumatological assessment as described elsewhere (see ref n. for the whole protocol).

### Sonographic assessment

Sonographic evaluations were performed by expert sonographers on 42 joints, 36 tendons, 12 entheses, and 2 bursae, using a scoring system specifically developed for PsA by the Ultrasound Study Group of the Italian Society for Rheumatology [14].

An experienced US physician was appointed at each participating centre to perform the scans, ensuring they were conducted without access to the patient's clinical data.

The US assessments were performed using a high-end US system equipped with multifrequency linear transducers and a high-frequency probe of at least 14 MHz.

The reliability of static image assessments was evaluated using kappa statistics (κ ≥ 0.7) to ensure substantial agreement. The results were compared with a ‘gold-standard’ scorer to validate the consistency of the scoring method. Details for the complete US protocol were previously published [8]

For the purpose of the study, we use the damage score from the ultrasound in psoriatic arthritis (UPsA) study calculated as follow: [(enthesophyte bc + entheseal calcification bc + irregular entheseal bone profile bc) × 1.62 + [(bone proliferation bc + enthesophyte bc) × 1.44] + [(joint erosion bc) × 1.04] + [tendon tear bc × 1.01] + [entheseal erosion bc × 1.00]. The UPsA damage score ranges from 0 to 10.

### Ethical approval

Written informed consent was obtained from all participants for study participation. The study received ethical approval from the institutional review boards of Santa Maria della Misericordia Hospital (Udine) and Cardarelli Hospital (Campobasso).

### Statistical analysis

Clinical and sonographic data were entered into Research Electronic Data Capture system. Analyses were performed using SPSS Statistics software (IBM) v. 26. Descriptive statistics (median, IQR, and percentages) were used to summarise patient characteristics. Statistical significance was determined using *P* values, with a threshold of <.05. Spearman rho correlation coefficient was used to evaluate possible correlations between UPsA damage score and clinical features including pain on numerical rating scale (NRS), Health Assessment Questionnaire Disability Index (HAQ-DI), DAPSA, PsA Impact of Disease, physician global assessment of disease activity on NRS (PhGA), patient global assessment of disease activity (PtGA) on NRS and ΔPtGA-PhGA.

## RESULTS

Of a total of 517 patients with PsA identified, 191 (36.9%) patients were on their second mechanism of action b/tsDMARDs. Of these, 53 (27.7%) met the criteria for D2T-PsA and were included in the analysis. Table 1 shows the clinical and US characteristics of the 53 D2T patients. Of these, 34 (64.1%) showed at least 1 site of active inflammation at US evaluation, whereas 19 showed no signs of active inflammation. The median DAPSA in the whole population is 25, with the majority of patients having high disease activity. The median (IQR) UPsA damage score of the 53 D2T patients was 3.79 (1.28-5.45). Notably, the UPsA damage score negatively correlates with tender joint count (rho –0.27, *P* = .04) but positively correlates with swollen joint count (rho +0.27, *P* = .05). Moreover, considering each item of the UpSA score, both the number of joint erosions and the number of joint osteophytes/osteoproliferation correlates positively with PhGA (rho +0.41, *P* = .002) (rho +0.27, *P* = .04) and correlates negatively with ΔPtGA-PhGA (rho –0.30, *P* = .02) (rho –0.31, *P* = .02) (Table 2). No significant correlation was detected among pain, PtGA, and HAQ-DI with UPsA damage score (figure in supplementary files). In patients without US signs of inflammation (*n* = 19), no significant correlation was found between each comparison. Finally, median (IQR) UPsA damage score was not significantly higher in patients with comorbidities (at least 1) and patients without comorbidities [3.9 (2.19-8.18) vs 2.9 (1-7.85); *P* = .16].

**Table 1:** Clinical characteristics of enrolled patients

Median (IQR); n (%)	D2T-PsA N = 53
Age (y)	59.0 (49.0, 65.0)
Female	34 (64)
Active smoker	7 (13)
BMI	26.9 (24.2, 30.5)
Family history of psoriatic arthritis	6 (11)
Comorbidities	
Obesity	19 (36)
Diabetes	11 (21)
Hypertension	19 (36)
Fatty liver disease	4 (7.5)
IBD	1 (1.9)
Depression	4 (7.5)
Malignancy	4 (7.5)
Hyperuricaemia	5 (9.4)
Cardiovascular disease	5 (9.4)
Dyslipidaemia	13 (25)
Charlson Comorbidity Index	2.0 (1.0, 3.0)
PASI	0.0 (0.0, 1.0)
Disease duration	11 (7, 23)
Predominant pattern of PsA	
Axial	3 (5,7)
Peripheral	50 (94,3)
Type of peripheral involvement	
Entheseal	6 (11.3)
Oligoarticular	19 (35.8)
Polyarticular	28 (52.9)
No of b/tsDMARDs failed	3.0 (2.0, 4.0)
No of mechanism of action failed	2.0 (2.0, 3.0)
No of cs/b/tsDMARDs not tolerated	0.0 (0.0, 1.0)
CRP (mg/dL)	0.3 (0.2, 0.9)
HAQ	1.0 (0.6, 1.5)
VAS Pain	7.5 (6.0, 8.0)
PhGA	5.0 (4.0, 7.0)
PtGA	7.0 (6.0, 8.0)
Δ PtGA-PhGA	1.0 (0.0, 4.0)
PASS, negative answer	45 (83)
DAPSA	25.0 (18.0, 38.0)
TJC	7.0 (2.0, 16.0)
SJC	1.0 (0.0, 3.0)
SPARCC	3.0 (1.0, 7.0)
Dactylitis	6 (11)
Fibromyalgia	14 (26)
Osteoarthritis	20 (38)
CSI value	47.0 (38.0, 54.0)
PSAID 12 value	5.8 (4.2, 6.8)
Total US damage score	3.79 (1.28-5.45)

cs/b/tsDMARDs, conventional synthetic/biologic/target synthetic Disease Modifying Anti-Rheumatic Drugs; BMI, body mass index; CRP, c-reactive protein; CSI, Central Sensitization Inventory; D2T-PsA, difficult-to-treat psoriatic arthritis; DAPSA, Disease Activity Index for Psoriatic Arthritis; HAQ, Health Assessment Questionnaire; IBD, inflammatory bowel disease; PASI, Psoriasis Area Severity Index; PASS, Patient Acceptable Symptom State; PhGA, physician global assessment; PSAID-12, Psoriatic Arthritis Impact of Disease; PtGA, patient global assessment; SJC, swollen joint count; SPARCC, Spondyloarthritis Research Consortium of Canada; TJC, tender joint count; US, ultrasound; VAS Pain, Visual Analogue Scale Pain.

**Table 2:** Correlations—Spearman rho (*P* value) between US Damage score and clinical characteristics of patients with D2T PsA

	TJC	SJC	Pain NRS	PhGA	PtGA	Delta (PtGA-PhGA)	DAPSA	HAQ-DI
US Damage Score	Rho –0.27 (0.04)	Rho +0.27 (0.05)	Rho –0.18 (0.18)	Rho +0.10 (0.45)	Rho –0.15 (0.27)	Rho –0.16 (0.24)	Rho –0.13 (0.34)	Rho +0.10 (0.47)
Number of entheseal calcifications count + entheseal bone irregularity count	Rho –0.26 (0.05)	Rho +0.16 (0.25)	Rho –0.13 (0.32)	Rho +0.01 (0.92)	Rho –0.11 (0.42)	Rho –0.05 (0.67)	Rho –0.16 (0.24)	Rho +0.09 (0.50)
Number of entheseal erosion count	Rho +0.05 (0.67)	Rho +0.24 (0.08)	Rho –0.13 (0.32)	Rho +0.14 (0.30)	Rho –0.19 (0.15)	Rho –0.18 (0.18)	Rho +0.12 (0.37)	Rho +0.03 (0.79)
Number of tendon lesion count	NP	NP	NP	NP	NP	NP	NP	NP
Number of joint erosion count	Rho +0.13 (0.34)	Rho +0.38 (0.04)	Rho +0.09 (0.51)	Rho +0.41 (0.02)	Rho +0.04 (0.74)	Rho –0.30 (0.03)	Rho +0.20 (0.14)	Rho +0.06 (0.67)
Number of joint osteoproliferation count + enthesophytes count	Rho –0.13 (0.33)	Rho +0.23 (0.09)	Rho –0.15 (0.25)	Rho +0.27 (0.04)	Rho –0.17 (0.20)	Rho –0.31 (0.02)	Rho –0.06 (0.63)	Rho +0.05 (0.71)

D2T, difficult to treat; DAPSA, Disease Activity Index for Psoriatic Arthritis; HAQ-DI, Health Assessment Questionnaire Disability Index; NRS, numerical rating scale; PhGA, physician global assessment of disease activity; PsA, psoriatic arthritis; PtGA, patient global assessment of disease activity; SJC, swollen joint count; TJC, tender joint count; US, ultrasound.

## DISCUSSION

Preliminary results of our study in patients with D2T PsA showed that UPsA damage score is associated with PhGA and swollen joints, but not with pain, suggesting that this symptom may be potentially driven by inflammation rather than damage itself.

A striking observation was that only two-thirds of patients with D2T-PsA (64.1%) demonstrated at least 1 site of active inflammation at US assessment, whereas the remaining one-third showed no sonographic evidence of inflammation. This reinforces the concept that persistent symptoms in PsA are not always attributable to active synovitis or enthesitis but may instead reflect noninflammatory mechanisms such as central sensitisation, structural damage, mechanical pain, or comorbid fibromyalgia [9].

In our study, the median UPsA damage score in our D2T cohort was 3.79, with a wide IQR, suggesting heterogeneous accrual of structural damage among patients. Interestingly, we observed a negative correlation between UPsA damage score and tender joint count, whereas a positive correlation was found with swollen joint count.

When analysing specific components of the UPsA score, the number of joint erosions and osteophytes/osteoproliferations correlated positively with PhGA but negatively with the delta between patient and physician global assessments (ΔPtGA–PhGA).

Notably, no significant correlation was found between pain scores or PtGA and the UPsA damage score. This dissociation may underscore the multifactorial nature of pain in PsA and highlight that structural joint changes alone cannot explain patient-reported outcomes. In fact, noninflammatory mechanisms such as central and peripheral sensitisation and neuropathic pain may be present in PsA and can drive pain and other patient-reported outcomes but with a dissociation with true inflammatory activity at the tissue level, which may be linked to progression of joint and entheseal damage [15]. Furthermore, despite the presence of comorbidities that may impact pain and other patient-reported outcomes, in our study, it does not seem to influence the UPsA score.

Our study extends this body of literature by concentrating specifically on D2T patients, a group underrepresented in imaging studies. The observed prevalence of damage and its modest correlations with clinical variables align with data from other cohorts showing that radiographic or sonographic damage progresses in a subset of patients despite therapy, and that clinical symptoms are only partially explained by structural pathology [16].

The strengths of our study include its multicentre design, blinded US assessments, and use of a validated scoring system to assess the presence joint and entheseal erosions, enthesophytes, joint osteoproliferation, and tendon tears. However, several limitations should be acknowledged. First, the cross-sectional design precludes causal inference and prevents evaluation of longitudinal associations between US findings and clinical outcomes. Second, the relatively small sample size of patients with D2T-PsA may have limited statistical power, particularly in subgroup analyses, and type I error cannot be excluded. In fact, the current data only show correlations at a single time point and do not allow conclusions regarding causality, temporal direction, or underlying mechanisms and further longitudinal studies with adequate power are needed to better explore this topic. Moreover, structural damage may have accumulated before the cross-sectional assessment; this limits the interpretation of our results. Finally, our study was designed before the definitions proposed by GRAPPA and EULAR and the applied definition is narrow and more treatment and DAPSA-focused compared with the multidimensional EULAR and GRAPPA criteria.

In conclusion, our study showed that D2T-PsA encompasses a heterogeneous spectrum of patients, of whom only a subset exhibits US-detectable active inflammation. The UPsA correlates modestly with physician-based assessments but is largely dissociated from patient-reported pain and global disease activity, underscoring the possible multifactorial nature of persistent symptoms. These findings highlight the necessity of an integrated, personalised approach to D2T-PsA that combines clinical, imaging, and patient-reported data.

## Competing interests

All authors declare they have no competing interests.

## Appendix Supplementary materials

Image, application 1

## Footnotes

Supplementary material associated with this article can be found in the online version at doi:10.1016/j.ero.2026.100217.
