# Supplementary material for: Does articular and entheseal structural damage drive pain and patient-driven reported outcomes in patients with difficult-to-treat psoriatic arthritis? Results from a cross-sectional study
Source: EULAR Rheumatol Open. 2026 Jul 16;2(3):100217. doi: 10.1016/j.ero.2026.100217 (PMC13650305; doi:10.1016/j.ero.2026.100217)
Supplement: Supplementary file 1 [file mmc1.docx]

A): Spearman rho correlation between Pain on numerical rating scale (NRS) and US damage score

Rho -0.18; p=0.18

B) Spearman rho correlation between patient global assessment of disease activity (PtGA) on numerical rating scale (NRS) and US damage score

Rho -0.15; p= 0.27

C) Spearman rho correlation between Health Assessment Questionnaire Disability Index (HAQ-DI) and US damage score

Rho +0.10; p=0.47
